# Cytoplasmic poly (A)-binding protein critically regulates epidermal maintenance and turnover in the planarian *Schmidtea mediterranea*

**DOI:** 10.1242/dev.152942

**Published:** 2017-09-01

**Authors:** Dhiru Bansal, Jahnavi Kulkarni, Kavana Nadahalli, Vairavan Lakshmanan, Srikar Krishna, Vidyanand Sasidharan, Jini Geo, Shilpa Dilipkumar, Renu Pasricha, Akash Gulyani, Srikala Raghavan, Dasaradhi Palakodeti

**Affiliations:** 1 Institute for Stem Cell Biology and Regenerative Medicine, GKVK PO, Bellary Road, Bangalore 560065, India; 2 Manipal University, Manipal 576104, India; 3 Transdisciplinary University, Bangalore 560064, India; 4 Sastra University, Thanjavur 613402 India; 5 National Centre for Biological Sciences, GKVK PO, Bellary Road, Bangalore 560065, India

**Keywords:** Planaria, Epidermis, Poly (A)-binding proteins, Neoblast, Regeneration

## Abstract

Identifying key cellular events that facilitate stem cell function and tissue organization is crucial for understanding the process of regeneration. Planarians are powerful model system to study regeneration and stem cell (neoblast) function. Here, using planaria, we show that the initial events of regeneration, such as epithelialization and epidermal organization are critically regulated by a novel cytoplasmic poly A-binding protein, SMED-PABPC2. Knockdown *of smed-pabpc2* leads to defects in epidermal lineage specification, disorganization of epidermis and ECM, and deregulated wound healing, resulting in the selective failure of neoblast proliferation near the wound region. Polysome profiling suggests that epidermal lineage transcripts, including *zfp-1*, are translationally regulated by SMED-PABPC2*.* Together, our results uncover a novel role for SMED-PABPC2 in the maintenance of epidermal and ECM integrity, critical for wound healing and subsequent processes for regeneration.

## Abstract (precis)

**Summary:** PABPC2 plays a crucial role in the maintenance of epidermal and extracellular matrix integrity, which is essential for neoblast function and regeneration in planarians.

## INTRODUCTION

Planarians have emerged as a tractable model system with which to study regeneration and stem cell biology. Their remarkable regenerative prowess comes from a population of adult somatic stem cells, called neoblasts, present throughout the mesenchyme of the animal (Baguñà, 2012; Wolff, 1962). Upon amputation or injury, neoblasts proliferate in two characteristic mitotic peaks in order to meet the demands of the regenerative process (Wenemoser and Reddien, 2010). The first mitotic peak is a body-wide response to injury and occurs within 12 h post-amputation (hpa) and declines after 24 hpa. The second mitotic peak is localized near the wound region and is observed at 48 hpa. Neoblast progeny initially form an undifferentiated, unpigmented tissue, called blastema, at the site of amputation, which is devoid of neoblast cells (Aboobaker, 2011; Rink, 2013). The blastema grows and differentiates further to form missing tissues and organs. Recent studies have also highlighted the role of non-neoblasts cells in instructing neoblasts for planarian regeneration. For example, position control genes (PCGs) that provide positional cues to neoblasts are expressed in the muscle cells (Witchley et al., 2013).

Recent efforts have uncovered broad roles for different post-transcriptional processes in stem cell/neoblast maintenance and differentiation (Guo et al., 2006; Palakodeti et al., 2008; Rouhana et al., 2010; Sasidharan et al., 2013; Solana et al., 2013; Lakshmanan et al., 2016; Wang et al., 2010). Here, we describe the role of cytoplasmic poly (A)-binding protein (PABPC) in neoblast function. PABPCs are RNA-binding proteins conserved across eukaryotes, and their role in translational regulation has been extensively studied (Gorgoni et al., 2011; Goss and Kleiman, 2013; Wang et al., 2010). However, PABPC also regulates mRNA turnover, nonsense-mediated decay and miRNA-mediated repression (Gorgoni et al., 2011; Goss and Kleiman, 2013).

Metazoans express multiple genes encoding PABPC: five in humans, three in *Xenopus*, two each in *C. elegans* and mouse, and one in yeast (Mangus et al., 2003). These genes are spatiotemporally regulated and have varied functions (Goss and Kleiman, 2013). In the planarian *Dugesia japonica*, two genes encoding cytoplasmic poly (A)-binding proteins, *dj-pabpc1* and *dj-pabpc2* have been reported. *dj-pabpc1* expression is ubiquitous and its knockdown leads to severe regeneration defects. Knockdown animals fail to form the blastema and lyse within 2 days post amputation (dpa) (Rouhana et al., 2010). However, the mechanism by which it regulates regeneration is not known. In *Schmidtea mediterranea*, *smed-pabpc1* is expressed in the germline tissue, and its knockdown leads to a block in meiotic progression, suggesting a role for SMED-PABPC1 in spermatogenesis (Wang et al., 2010)*.* Our study identified an additional cytoplasmic poly (A)-binding protein, *smed-pabpc2* (referred to as *pabpc2*) from the *Schmidtea mediterranea* transcriptome (Resch et al., 2012), which is a homologue of *dj-pabpc1*.

*pabpc2* is expressed in majority of the cell types, apart from the pharynx and terminally differentiated epidermal cells (*NB.22.1e^+^* and *laminB^+^*). Knockdown of *pabpc2* leads to drastic regeneration and homeostatic defects in *Schmidtea mediterranea*. As PABPC in eukaryotes is a known translation initiator, we performed polysome profiling followed by transcriptome sequencing from control and knockdown animals to identify the targets of PABPC2. Strikingly, a specific set of transcripts that are essential for epidermal lineage determination, including *zfp-1*, were translationally repressed in knockdown animals. Extensive molecular characterization of *pabpc2* knockdown phenotypes revealed defects in the epidermal turnover that led to defective organization of the epidermal tissue along with the loss of extracellular matrix (ECM) integrity. Further analysis showed that the epidermal defects in knockdown animals led to sustained injury response, which subsequently resulted in the failure of activation of the second mitotic peak that is essential for blastema formation. Taken together, our data points to a crucial role for PABPC2 in the maintenance of epidermal and ECM integrity, which in turn is essential for neoblast function and regeneration in planarians.

## RESULTS

### *pabpc2* showed enriched expression in epidermal lineage, gut and neoblasts

**Fig. 1.**
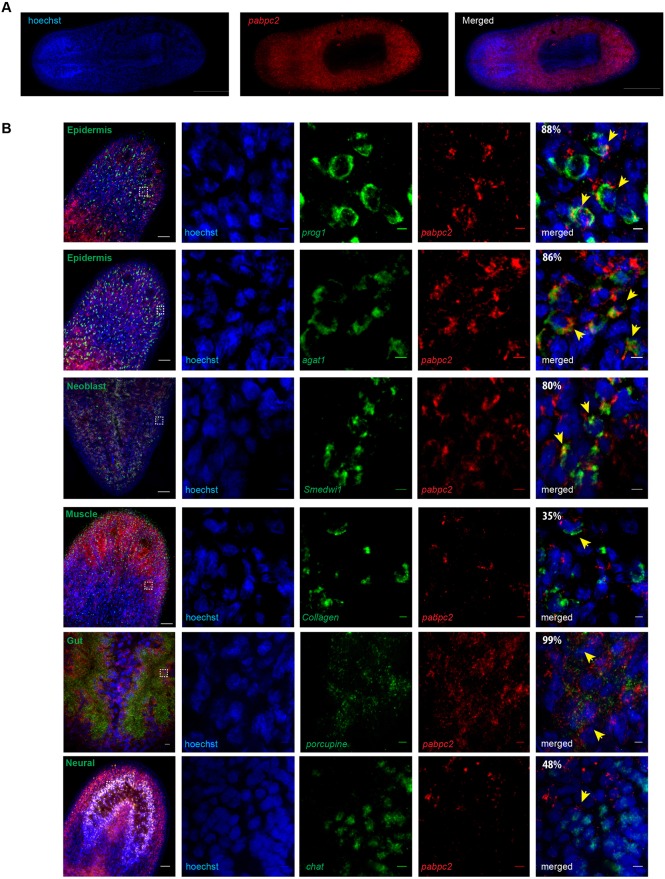
**Identification and characterization of SMED-PABPC2.** (A) Fluorescent *in situ* hybridization to study the expression pattern of *smed-pabpc2.* Scale bars: 500 μm. (B) Double fluorescent *in situ* hybridization showing co-expression of *pabpc2* with *prog1*, *agat1*, *smedwi1*, *collagen*, *porcupine* and *chat*. The images in the first column were taken at 20× magnification. Scale bars: 50 μm. The white boxes indicate the area magnified in the columns to the right. The percentage of colocalization is shown in the last panel. Probes are indicated; yellow arrows indicate co-labeled cells. Scale bars: 5 μm. *n*=6. See also Fig. S1.

Smed-PABPC2 protein has well-conserved RRM domains and shows 44% identity to mammalian cytoplasmic poly (A)-binding protein 1 (Fig. S1A,B). Fluorescent *in situ* hybridization to study the expression pattern revealed that *pabpc2* is mostly expressed in all the cell types of planarians, except in the pharynx (Fig. 1A). Fluorescent *in situ* hybridization on *pabpc2* knockdown animals showed the absence of *pabpc2* staining, suggesting that the *pabpc2* probe is specific (Fig. S1C). We then investigated the expression of *pabpc2* in different cell types from the available single cell sequencing (SCS) data (https://radiant.wi.mit.edu/app/), which identified transcripts enriched in different cell types (Wurtzel et al., 2015). In general, *pabpc2* expression is observed in all the cell types, but is enriched in the epidermal lineage, gut and neoblasts (Fig. S1D). The expression of *pabpc2* in different cell lineages was also confirmed by colocalization studies using lineage and tissue-specific markers for epidermal lineage (*prog1*, *agat1*), brain (*chat*), gut (*porcupine*), muscle (*collagen*), and neoblast (*smedwi1*). Co-expression studies revealed that 88%, 86%, 80% and 99% of *pabpc2*^+^ cells colocalized with *prog1*, *agat1*, *smedwi1* and *porcupine*, respectively (Fig. 1B). However, 35% and 48% of the *pabpc2*^+^ cells colocalized with *collagen* and *chat*, respectively, confirming the single-cell sequencing data, which showed enriched expression of *pabpc2* in the epidermal lineage, gut and neoblasts. Interestingly, *pabpc2* did not colocalize with terminally differentiated epidermal cells (*laminB^+^* and *Nb.22.1e^+^*) (Fig. S1E). Next, we investigated its functional role in planarian regeneration and homeostasis.

### *pabpc2* is essential for planarian regeneration and homeostasis

**Fig. 2.**
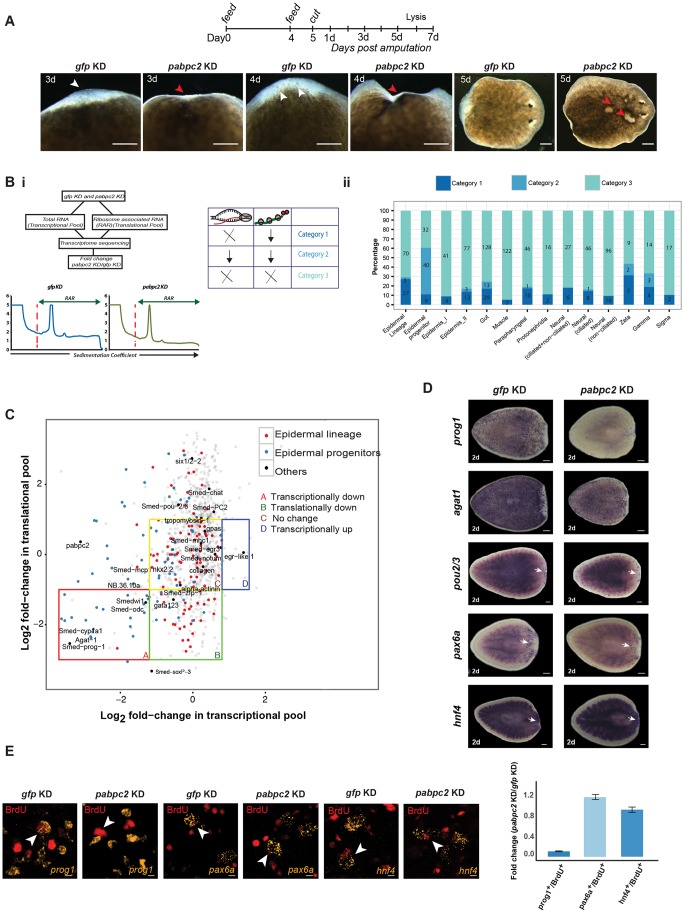
**PABPC2 regulates epidermal lineage transcripts.** (A) Timeline showing RNAi feed schedule. Images were taken at 3, 4 and 5 dpa after dsRNA treatment. White arrowheads show normal blastema in control animals. Red arrowheads highlight the defective blastema and the lesions in knockdown animals (100/100). Scale bars: 200 μm. (Bi) The method used to identify the transcripts associated with the ribosome and cellular transcripts in the *gfp* and *pabpc2* knockdown animals. ×, no change; ↓, decrease. (Bii) Stack bar depicting number and percentage of transcripts that belong to different categories across various cell types. Fold change was calculated by taking the ratio of the normalized number of transcripts between *pabpc2* and *gfp* knockdown animals. (C) Scatter plot of fold-change values (PABPC/GFP) showing the distribution of transcripts between the transcriptional and translational pool. Transcripts from different categories are marked as four quadrants A, B, C and D. Transcripts that belong to epidermal lineage and epidermal progenitors are highlighted in red and blue, respectively. Some well-known markers belonging to different cell types and wound-healing genes are labeled in the scatter plot. (D) Whole-mount *in situ* hybridization using different progenitor markers such as *prog1*, *agat1*, *pou2/3*, *hnf4* and *pax6a* at 2 dpa in *gfp* and *pabpc2* knockdown animals. Epidermal progenitors (*prog1* and *agat1*) showed a significant reduction in the expression upon *pabpc2* knockdown, unlike other progenitors (*hnf4*, *pax6a* and *pou2/3*). White arrows indicate staining in the blastema. Scale bars: 200 μm (*n*=10). (E) Confocal images showing BrdU and progenitor (*prog1*, *hnf4* and *pax6a*)*-*positive cells in *gfp* and *pabpc2* knockdown animals. BrdU was injected post-2nd feed and animals were fixed 2 days post-BrdU injections. Equal numbers of BrdU cells were counted in *gfp* and *pabpc2* knockdown animals and the numbers of colocalized cells were counted for each progenitor. The histogram depicts the fold change in colocalized cells in *gfp* and *pabpc2* knockdown animals. Error bars were calculated from biological replicates. Yellow arrows indicate co-labeled cells. Scale bars: 5 μm. *n*=6. See also Fig. S2.

Bacteria expressing *pabpc2* dsRNA were fed to the animals for knockdown of the gene. Green fluorescent protein (*gfp*) dsRNA was used as a negative control. Knockdown of *pabpc2* in uncut animals resulted in the formation of lesions within 5-6 days post-2nd feed and the animals underwent lysis by day 11 (Fig. S2A). In regenerating animals, *pabpc2* knockdown resulted in a smaller blastema that regressed by 3-4 dpa and the animals subsequently lysed by 5-7 dpa (Fig. 2A). The control animals (*gfp* dsRNA-treated animals) showed no defects even after 3 weeks post-feeding. In summary, these results clearly show that *pabpc2* is crucial for homeostasis and regeneration in planarians.

### *pabpc2* regulates transcripts essential for epidermal lineage during planarian regeneration

PABPCs enhance translation initiation by interacting with the translation initiation factor eIF4G and form a ‘closed loop’ mRNA conformation that is essential for the ribosome assembly (Tarun and Sachs, 1996). To identify the transcripts that were translationally repressed upon *pabpc2* knockdown during regeneration, ribosome-associated RNA (RAR) was analyzed at 24 hpa in control and *pabpc2* knockdown animals. mRNA from the ribosomal complexes (monosomes and polysomes) was purified, and deep sequencing was performed to identify the transcripts depleted from the translational pool in *pabpc2* knockdown animals. We also performed deep sequencing of cellular mRNA from the control and knockdown animals at 24 hpa to identify the transcripts that were transcribed but not translated (Fig. 2Bi).

The reads were mapped to the transcriptome database (see Materials and Methods) to identify the transcripts that were translationally repressed in *pabpc2* knockdown animals. A total of 4412 transcripts with an adjusted *P*-value of less than 0.05 and a minimum of 10 reads mapping to each of the transcripts after normalization were considered for further analysis (Table S1). Recent efforts based on the single-cell transcriptome analysis assigned 4787 transcripts to 13 different cell types from planaria (Wurtzel et al., 2015). From our transcriptome analysis, we identified 991 of these 4787 transcripts with coverage for each cell type varying from ∼6 to 44% (Fig. S2B).

The transcripts were classified into three broad categories based on a change of at least twofold calculated between the *pabpc2* knockdown and mock-treated animals. The categories include: (1) transcripts that were translationally downregulated; (2) transcripts that were transcriptionally downregulated; and (3) transcripts that were either unaffected or upregulated both transcriptionally and translationally. We observed that 462 out of 991 (46.7%) transcripts fell into the 3rd category, representing all cell types (Fig. 2Bii). This suggests that PABPC2 is not a global regulator of translation in planaria but regulates translation of specific transcripts.

In *pabpc2* knockdown animals, category 1 transcripts (actively transcribed but not translated) were found in all the cell types to varying extents (Fig. 2Bii). The majority of these transcripts belonged to epidermal lineage (27%) followed by gut (17%). In spite of the low coverage (10%) of epidermal lineage transcripts from our transcriptome pool (Fig. S2B), the majority of these transcripts belonged to category 1, suggesting that PABPC2 could potentially be an active regulator of epidermal lineage transcripts. As further proof, we also found that among different classes of the neoblast population, the majority of transcripts in the category 1 belonged to the zeta class (essential for epidermal lineage) and gamma class (essential for gut lineage). Some of the epidermal lineage transcripts depleted from the translational pool include *gata123*, *soxP-3* and *zfp-1*, which are essential for the epidermal lineage formation (Fig. 2C) (van Wolfswinkel et al., 2014). Next, we probed the nature of the translationally repressed transcripts belonging to other cell types, such as neural, gut, muscle and protonephridia. Interestingly, none of the well-characterized transcripts essential for neural (*chat*, *PC2*, *gpas*), gut (*nkx 2.2*), muscle (*tropomyosin*, *smed-mhc-1*) and protonephridia (*pou2/3*, *rootletin*, *six1/2-2*) specification was affected (Fig. 2C) (Forsthoefel et al., 2012; Scimone et al., 2014; Witchley et al., 2013).

We also investigated the transcripts belonging to category 2, which were transcriptionally downregulated. Strikingly, majority of the category 2 transcripts belonged to either epidermal progenitors (49.4%) or gut cells (7.6%) (Fig. 2Bii). Some of the well-characterized epidermal progenitor markers such as *prog-1*, *agat-1*, *odc* and *cyp1a1* were transcriptionally downregulated (Fig. 2C).

The RAR data suggest that in *pabpc2* knockdown animals, the *zfp-1* translation was defective but the transcript levels remain unchanged, whereas the *prog1* and *agat1* transcript levels were significantly reduced (Fig. 2C). We confirmed these results by real-time PCR on control and knockdown animals at 24 hpa and we found no change in overall *zfp-1* transcript level (Fig. S2C). However, the levels of *prog1* and *agat1* were dramatically reduced (Fig. S2D). Here, we assume that the decrease in levels of transcripts such as *zfp-1* from the translation pool subsequently leads to the failure of the formation of epidermal progenitors in the knockdown animals. Interestingly, the transcripts from other cell types that were translationally downregulated did not result in overt phenotypes.

To validate the RAR data, which showed the potential role of PABPC2 in epidermal lineage formation, we performed whole-mount *in situ* hybridization on *gfp* and *pabpc2* knockdown animals with several known progenitors for epidermis (*prog1*, *agat1*), gut (*hnf4*), neurons (*pax6a*) and protonephridia (*pou2*/3) at 2 dpa. In accordance with RAR and transcriptome data, we observed a dramatic decrease only in the epidermal progenitors in the knockdown animals (Fig. 2D). To further confirm the crucial role of PABPC2 in the differentiation of neoblasts into epidermal progenitors, we also performed BrdU labeling on uncut control and knockdown animals. We administered BrdU at 24 h post-2nd feeding and fixed the animals after 2 days. Around a 90% decrease in the formation of new epidermal progenitor cells was observed in *pabpc2* knockdown animals compared with control. However, no defect was observed in the formation of progenitors of gut and brain (Fig. 2E). Taken together, our results demonstrate the role of PABPC2 in the generation of epidermal lineage, crucial for both epidermal regeneration and turnover.

### *pabpc2* knockdown animals have disorganized epidermal tissue and extracellular matrix

**Fig. 3.**
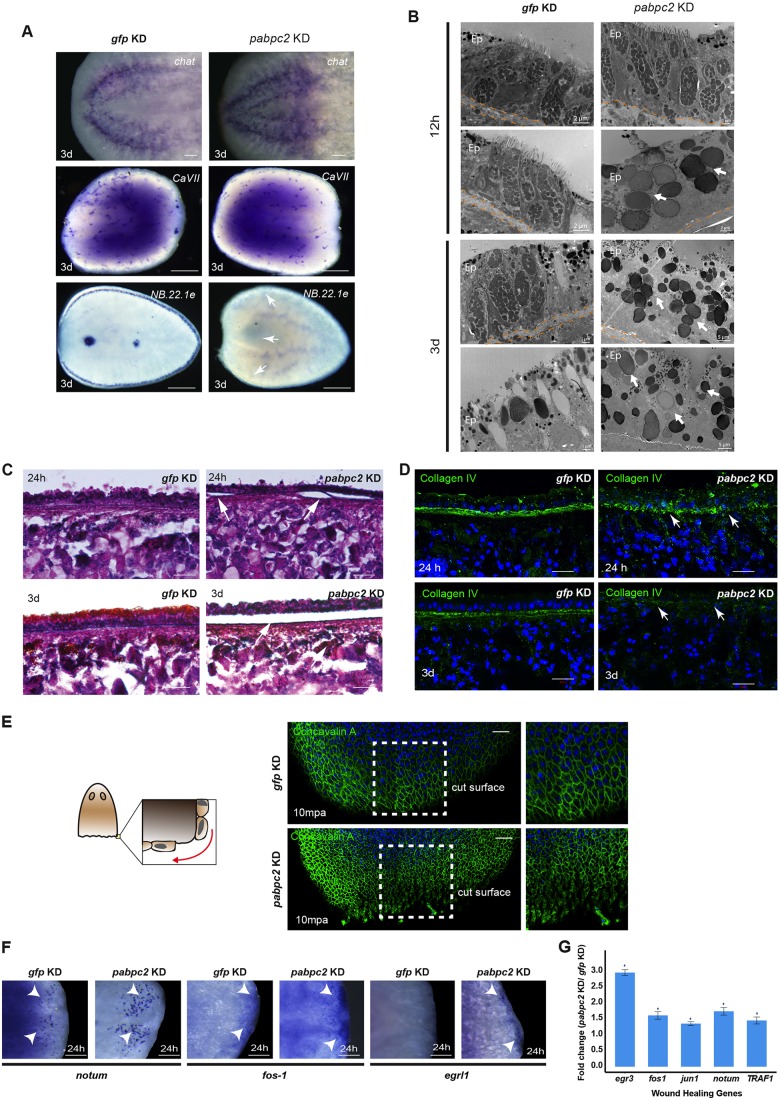
**SMED-PABPC2 is essential for maintaining epidermal and ECM integrity and epithelialization.** (A) Whole-mount *in situ* hybridization showing expression of the differentiated tissue markers *chat*, *cavII* and *NB.22.1e* in *gfp* and *pabpc2* knockdown animals. Scale bars: 500 μm. White arrows indicate loss of *NB.22.1e* expression in *pabpc2* knockdown animals. *n*=10. (B) EM images showing organization of the epidermal tissue on the non-regenerating side at 12 hpa and 3 dpa in *gfp* and *pabpc2* knockdown animals. Arrows show rhabdite-like cells in knockdown animals. Ep, epidermis. *n*=5. (C) Histological sections showing the organization of epidermis after *pabpc2* knockdown in regenerating animals. Sagittal sections made from the regenerating animals at 24 h and 3 days post-amputation were stained with Hematoxylin and Eosin. Arrows show peeling of the epidermis in *pabpc2* knockdown animals in regions away from amputation. Scale bars: 20 µm. *n*=9. (D) Maximum intensity projections of *z*-stacks of *gfp* and *pabpc2* knockdown sagittal sections stained with collagen IV antibody at 24 h and 3 days post amputation. Arrows showing disorganization of ECM in *pabpc2* knockdown animals. Scale bars: 20 µm. *n*=10. (E) Schematic showing stretching of epithelial cells near the wound region. Confocal images of *gfp* and *pabpc2* knockdown animals stained with concavalin A*-*FITC showing the organization of dorsal epidermal tissue near the amputated region at 10 min post-amputation (mpa). The image is tiled. Scale bars: 100 µm. *n*=5. (F) Whole-mount *in situ* hybridization showing upregulation of early wound-healing genes such as *notum*, *fos-1* and *egr like 1* (*egrl1*) near the blastema at 24 hpa in *pabpc2* knockdown animals. Scale bars: 50 µm. *n*=10. Arrowheads show the expression of transcripts in the blastema region. (G) Quantification of level of expression of wound-healing genes by qRT-PCR. Fold-change of wound-healing gene levels in *pabpc2* knockdown animals at 24 hpa. The error bars are drawn from biological triplicates and indicate s.e.m. *P<0.05. See also Fig. S3.

Our results based on the expression pattern and molecular function of PABPC2 categorically highlight its role in epidermal maintenance and/or turnover. To further confirm this, we investigated the effect of *pabpc2* knockdown on the epidermal tissue. Whole-mount *in situ* hybridization on control and *pabpc2* knockdown animals at 3 dpa for the epidermal marker *NB.22.1e*, which marks marginal adhesive glands and ventral mouth opening (Reddien et al., 2007; Tazaki et al., 2002), revealed a complete absence of its expression, suggesting a drastic defect in the epidermal tissue. In contrast, other tissue-specific markers, such as *cavII*, which marks the tubules of the protonephridia, and *chat*, which marks the bilobed structure and the ventral nerve cord of CNS (Rink et al., 2011; van Wolfswinkel et al., 2014; Wagner et al., 2011), showed normal expression patterns in both control and knockdown animals (Fig. 3A). Furthermore, we also validated the disorganization of epidermal cells in knockdown animals by immunostaining with anti-rootletin antibody, which marks the ciliated epidermal cells. In control animals, immunostaining revealed a concentric organization of rootlets in the ciliated epidermal cells. However, in the knockdown animals the organization of the rootlets was disrupted, revealing the abnormal organization of the epidermal cells (Fig. S3A).

Epidermal tissue is primarily made up of two cell types: epithelial cells and gland cells. Interspersed between the epithelial cells are the gland cells that contain rod-shaped secretory granules called rhabdites. Studies have shown that rhabdite-forming cells contribute to the successive renewal of epidermal cells (Hori, 1979; Skaer, 1965). Rhabdites are formed in the mesenchyme and migrate to the epidermal tissue through ducts of the gland cells. Once in the epidermis, they are rapidly released out through the exterior opening of the gland cells to form the mucous layer (Martin, 1978). Electron microscopy (EM) studies to investigate the organization of epidermal tissue in *pabpc2* knockdown revealed disorganization of epidermal tissue as early as 12 hpa in knockdown animals (Fig. 3B). The knockdown animals lacked epithelial cells and had rhabdite-like bodies throughout the epidermis, which showed a dramatic increase by 3 dpa (Fig. 3B). Similar results were also observed in the uncut knockdown animals (Fig. S3B).

The defect in the epidermal tissue in the knockdown animals is potentially due to the failure in the generation of the epidermal progenitors. This was supported by the whole-mount *in situ* hybridization carried out in *pabpc2* knockdown animals as early as 6 hpa, which showed decrease in the epidermal progenitors (Fig. S2D). We further tested the sensitivity of epidermal progenitor formation to the PABPC2 levels by feeding low doses of *pabpc2* dsRNA to the animals (one dose of dsRNA feed). The animals fed with low dose of dsRNA showed significant decrease in the *prog1^+^* and *agat1^+^* cells (Fig. S3C), suggesting that PABPC2 is crucial for the epidermal progenitor formation. In planarians, the epidermal layer is attached to the sub-epidermal tissue via an extracellular matrix (ECM) (Hori, 1979; Hori, 1991). As *pabpc2* knockdown disrupts organization of the epidermal cells, we also investigated the attachment of epidermal tissue to the ECM in the knockdown animals. Sagittal sections stained with Hematoxylin and Eosin revealed severe blistering and detachment of the epidermis from the underlying ECM in knockdown animals as early as 24 hpa, which dramatically increased by 3 dpa (Fig. 3C). We next investigated whether the epidermal detachment could be due to the disorganization of the ECM using collagen as a marker. Collagen is the major component of the ECM and is secreted by the epidermal cells in planaria (Hori, 1980). Mammalian collagen IV antibody, which crossreacts with the planarian collagen in the ECM, was used to study the ECM organization. The control animals showed an intact, closely packed collagen staining between epidermal and sub-epidermal layers. However, *pabpc2* knockdown animals showed diffused staining by 24 hpa, which was spread across epidermal and sub-epidermal layer by 3 dpa (Fig. 3D). These results clearly demonstrate that PABPC2 is essential for the maintenance of epidermis and ECM integrity.

### *pabpc2* knockdown animals showed sustained wound response during regeneration and homeostasis

Next, we investigated the role of PABPC2 in wound healing because of its requirement for the maintenance of the epidermal tissue. In planarians, it has been reported that within 10-15 min post-amputation (mpa) epidermal cells near the injury site close the wound as a result of muscle contraction (Chandebois, 1980). This is followed by passive stretching of the pre-existing epithelial cells near the wound region to form a thin film of cells (Chandebois, 1980; Morita et al., 1969). However, the importance of the epidermis in wound closure and its impact on the stem cell function has not been elucidated. The epidermal organization near the cut surface in *pabpc2* knockdown animals was studied by staining the animals with the lectin concavalin A, which marks the boundaries of the epidermal cells (Zayas et al., 2010). In the control treated animals, the epidermal cells near the wound region were elongated and subsequently lead to the closure of the wound (Fig. 3E, Fig. S3D). Interestingly in *pabpc2* knockdown animals, pre-existing epithelial cells near the wound region failed to stretch and showed a rounded appearance as early as 10 mpa (minutes post-amputation) (Fig. 3E). This defect in the knockdown animals persisted at a later time point, 24 hpa (Fig. S3D), suggesting a defect in the wound closure.

We reasoned that the knockdown animals might sense the epithelialization defect as a sustained injury leading to the deregulation of some of the early wound-response genes. Whole-mount *in situ* hybridization analysis of *pabpc2* knockdown animals indeed showed an upregulation of early wound-response genes such as *notum* and *fos-1* at 24 hpa (Fig. 3F) compared with the control animals. In addition, *egr like 1* (*egrl1*), an early wound-response gene, the expression of which is normally seen until 1 hpa (Wenemoser et al., 2012), showed sustained expression even at 24 hpa in *pabpc2* knockdown animals (Fig. 3F). qRT-PCR analysis on some of the early wound healing further corroborated with whole-mount *in situ* hybridization (Fig. 3G). Interestingly, *pabpc2* knockdown animals that were not injured or amputated also showed expression of wound healing genes such as *jun-1* and *fos-1*, which was not observed in the control uncut animals (Fig. S3E). Furthermore, the transcriptome and qRT-PCR analysis also validated the upregulation of early wound-response genes such as *egrl1*, *notum*, *TRAF1* and *egr3* in the *pabpc2* knockdown animals (Fig. S3F,G). Taken together, these results suggest a crucial role for PABPC2 in the organization of epidermal tissue near the site of injury that is essential for the wound response.

### *pabpc2* knockdown leads to a defect in neoblast proliferation near the wound region

**Fig. 4.**
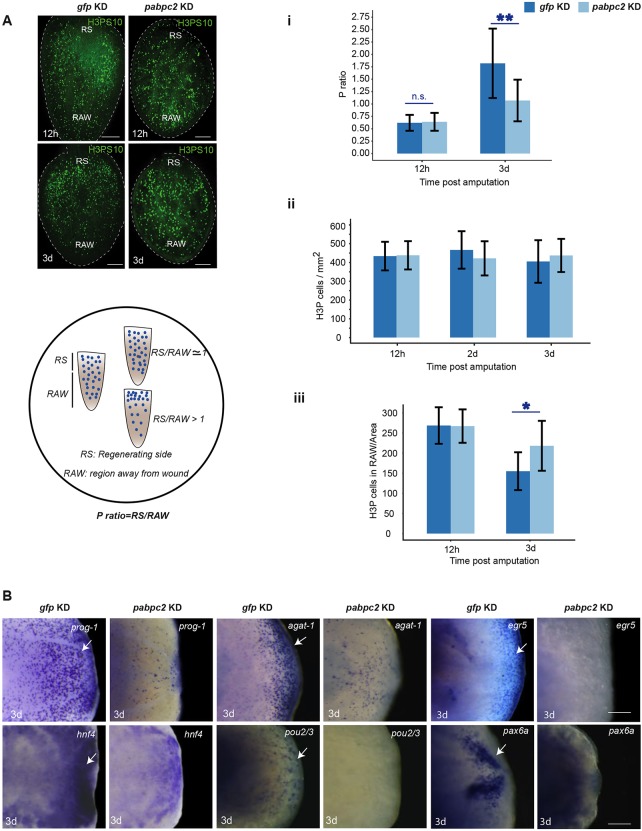
**Effect of *smed-pabpc2* knockdown on neoblast proliferation and blastema formation.** (A) Max intensity projections of confocal images showing H3PS10^+^ cells in regenerating animals in *gfp* and *pabpc2* knockdown animals. Scale bars: 100 µm. *n*=21. Schematic showing the procedure of calculating the P-ratio. Animals were divided in the ratio of 1:2 from the cut side and considered as regenerating side (RS) and region away from wound (RAW), respectively. Mitotic cells were calculated in both the regions and normalized to per unit animal area. P ratio=RS/RAW. (i) The P ratio at 12 hpa and 3 dpa in *gfp* and *pabpc2* knockdown animals. *pabpc2* knockdown animals showed a P ratio close to 1 even at 3 dpa, unlike control animals. The difference between the P ratios in control and knockdown animals at 3 dpa was significant (**P<0.0001). n.s., non-significant. (ii) The total number of mitotic cells in *gfp and smed-pabpc2* knockdown animals at 12 hpa, 2 dpa and 3 dpa. (iii) Cell numbers in the region away from the wound at 12 hpa and 3 dpa. A significant increase was observed in cell number in RAW in *pabpc2* knockdown animals at 3 dpa (*P<0.05) (*n*=21). (B) Whole-mount *in situ* hybridization showing expression of progenitor markers, *prog-1*, *agat-1*, *egr5*, *hnf4*, *pax6A* and *pou2/3* in *pabpc2* knockdown and *gfp* knockdown animals at 3 dpa. White arrows indicate the staining observed in the blastema. Scale bars: 50 μm. *n*=10. See also Fig. S4.

Neoblast proliferation in *Schmidtea mediterranea* occurs in two distinct phases during regeneration: first, body-wide proliferation at 4-12 hpa; and second, specific proliferation near the amputated region, which occurs at 2-3 dpa (Wenemoser and Reddien, 2010). The second mitotic phase is crucial for blastema formation. As *pabpc2* knockdown animals showed regression of the blastema, we investigated the status of the two mitotic peaks during regeneration in the control and *pabpc2* knockdown animals. The changes in the two mitotic peaks can be measured by calculating the ratio of the number of proliferating cells (H3PS10 stained) near the wound region compared with away from the wound region per unit area (P-ratio). A P-ratio close to one indicates the first mitotic peak, which is a body-wide response because of the even distribution of proliferating neoblasts across the regenerating animal. A P-ratio greater than one indicates second mitotic peak, because of the increased number of neoblasts present near the wound region (Fig. 4A). We measured the P-ratios at 12 hpa (first mitotic peak) and 3 dpa (second mitotic peak) in *pabpc2* and *gfp* knockdown animals. The P-ratio was close to one at 12 hpa in both *gfp* and *pabpc2* knockdown animals, suggesting normal activation of the first mitotic peak (Fig. 4Ai). Interestingly, the P-ratio remained close to one at 3 dpa in *pabpc2* knockdown animals, whereas the P-ratio in the control animals was greater than one, indicating that PABPC*2* is crucial for the second mitotic peak in the regenerating animals (Fig. 4Ai). However, there was no significant reduction in the overall number of the H3P^+^ cells, suggesting that *pabpc2* knockdown has no effect on the overall proliferation of neoblasts upon amputation (Fig. 4Aii). We also performed whole-mount *in situ* hybridization with a neoblast marker, *smedwi-1*, at 3 dpa and found no observable changes in the expression of *smedwi-1* (Fig. S4A). We predicted that the reduction in the number of H3P^+^ cells near the wound region would be compensated for by the increase in the number of dividing cells away from the wound region. This prediction was tested by counting the number of H3P^+^ cells in the region away from the wound site. We observed a 1.4-fold (*P*≤0.001) increase in the number of mitotic cells away from the wound region in *pabpc2* knockdown animals at 3 dpa (Fig. 4Aiii). This suggests that, although the total number of neoblasts in *pabpc2* knockdown animals is similar to control animals, the defect in mitosis near the wound region is compensated for by an increase in mitosis away from the wound region in knockdown animals. Taken together, the data presented clearly demonstrate that the knockdown of *pabpc2* leads to the defect in the localized proliferation of neoblasts near the wound region, resulting in the regression of the blastema.

Our previous result showed that the knockdown of *pabpc2* in the uncut animals led to the expression of early wound-response genes, suggesting a sustained injury throughout the animals. The sustained early wound response has been implicated in the prolonged global proliferation in the regeneration animals (Lin and Pearson, 2017). We predicted that the rate of neoblast proliferation would increase in the uncut animals after *pabpc2* knockdown due to the sustained expression of early wound-response genes. We indeed found 1.5-fold increase in the number of H3P^+^ cells in the knockdown animals compared with the control animals (Fig. S4B). The sustained expression of the early wound-response genes in the regenerating animals could possibly explain the prolonged global proliferation and a failure in localized proliferation.

### *pabpc2* knockdown animals show subsequent loss of progenitors in the blastema

In regenerating planarians, the bimodal pattern of neoblast proliferation was shown to be crucial for blastema formation (Wenemoser and Reddien, 2010). The regenerating blastema is typically characterized by the presence of neoblast progenitors (Rink, 2013). As we observed a failure in the localized proliferation of neoblasts leading to a defective blastema formation, we investigated the presence of several known progenitors near the wound region at 3 dpa in *gfp* and *pabpc2* knockdown animals. Whole-mount *in situ* hybridization for epidermal progenitors (*prog1*, *agat1* and *egr5*) (Tu et al., 2015), and progenitors of other tissues, such as gut (*hnf4*), protonephridia (*pou2*/3) and brain (*pax6A*) (Scimone et al., 2011, 2014; Wagner et al., 2011) showed a drastic reduction in their expression at 3 dpa near the blastema (Fig. 4B). Together, these results confirm that *pabpc2* knockdown animals, which showed failure in the localized proliferation, had a defective blastema.

### *pabpc2* knockdown animals showed no gross defects in sub-epidermal muscle layer but failed to express PCGs at 3 dpa

**Fig. 5.**
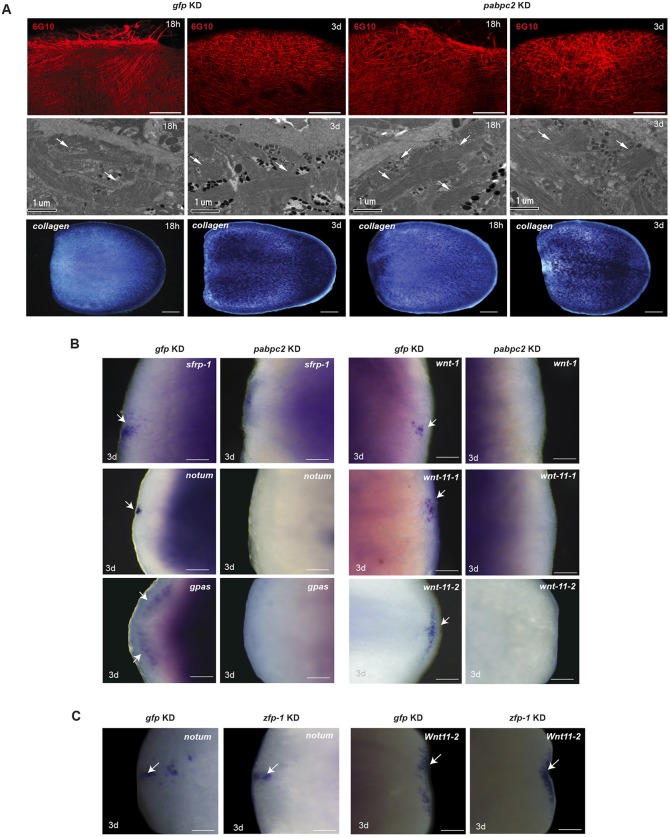
***smed-pabpc2* does not affect sub-epidermal muscle cells but affects PCGs expression.** (A) Confocal images showing the organization of muscle cells stained with anti-6G10 antibody near the blastema after 18 h and 3 dpa in *gfp* and *pabpc2* knockdown animals. Images were taken using LSM700 confocal microscope. Scale bars: 100 µm. *n*=6. EM images and whole-mount *in situ* hybridization with *collagen* showing muscle organization in *gfp* and *pabpc2* knockdown animals at 18 hpa and 3 dpa. Arrows indicate sub-epidermal muscle cells. Scale bars: 200 µm. *n*=5. (B) Whole-mount *in situ* hybridization showing expression of anterior (*sfrp-1*, *notum* and *gpas*) and posterior (*wnt-1*, *wnt11-1* and *wnt11-2*) PCGs in control and *pabpc2* knockdown animals in the blastema at 3 dpa. Arrows mark the expression of PCGs in the blastema. Scale bars: 50 µm. (C) Whole-mount *in situ* hybridization showing expression of *notum* and *wnt11-2* in *zfp-1* knockdown animals at 3 dpa in the blastema region. Unlike *pabpc2* knockdown animals, *zfp-1* knockdown animals expressed PCGs in the blastema. Scale bars: 50 µm. *n*=8. Arrows indicate PCG expression in the blastema.

The pronounced defect in ECM and epidermis in *pabpc2* knockdown animals led us to investigate the effect of *pabpc2* knockdown on sub-epidermal cells. Planarian sub-epidermal layer is made up of circular, longitudinal and diagonal fibers that form the muscle net (Cebrià et al., 1997). We used 6G10 antibody that marks muscle fibers to study the organization of sub-epidermal muscle cells (Ross et al., 2015). Immunostaining of the control and *pabpc2* knockdown animals showed no gross defect in the muscle organization (Fig. 5A). This result was further corroborated by the EM studies, which showed the normal appearance of muscles in the sub-epidermal layers (Fig. 5A). Whole-mount *in situ* hybridization to study the expression pattern of *collagen*, which is a marker for the muscle cells, also showed no significant changes at 18 hpa and 3 dpa in control and *pabpc2* knockdown animals (Fig. 5A). We also investigated the expression of *collagen* in uncut *pabpc2* knockdown animals and found no observable change (Fig. S5A). Thus, our results suggest that the knockdown of *pabpc2* did not have a profound effect on the organization of the sub-epidermal muscle layer in planaria.

In planarians, the sub-epidermal muscle layer expresses most of the PCGs that provide positional cues essential for neoblast differentiation (Witchley et al., 2013). We investigated the expression of the PCGs such as *notum*, *sfrp* and *gpas*, which specify anteriority, and *wnt1*, *wnt11*-*1* and *wnt11-2*, which specify posteriority (Gurley et al., 2010; Mii and Taira, 2009; Petersen and Reddien, 2011). Whole-mount *in situ* hybridization on *pabpc2* knockdown animals at 3 dpa showed a complete absence of PCG expression both in the anterior and posterior regenerating tissue (blastema) (Fig. 5B). The reduction in the expression of PCGs could be either due to the disorganization of epidermal tissue or to their translational downregulation resulting from *pabpc2* knockdown. We tested both these possibilities by knockdown of *zfp-1* and by transcriptome sequencing of RAR. The *zfp-1* knockdown animals, which have perturbed epidermal organization, did not show any loss of PCG expression (*wnt11-1* and *notum*) at 3 dpa (Fig. 5C), suggesting that the disruption of epidermal organization per se does not affect PCG expression. We also investigated the translational status of the PCGs in *pabpc2* knockdown animals. The transcriptome sequencing of RAR revealed more than twofold upregulation in the association of the PCG transcripts (*wnt11-5*, *sfrp3*, *evi/WIs* and *slit1*) with the translational pool in the knockdown animals at 24 hpa. This suggests that the PABPC2 is not required for the translation of PCG transcripts (Fig. S5B). Although we observed upregulation of *notum* at 24 hpa in the knockdown animals, its expression was subsequently downregulated by 3 dpa (Fig. 3F). It is very well known that some of the PCGs encoding members of Wnt and TGFβ signaling pathways are also wound-response genes that are expressed after 6 hpa (Wenemoser et al., 2012). As *pabpc2* knockdown animals have sustained expression of early wound-response genes, the absence of PCG expression seen at 3 dpa could be an effect of deregulated wound response*.*

In *pabpc2* knockdown animals, we also observed disorganization of the ECM, which was not observed in the *zfp-1* knockdown animals (Fig. S5C). Here, we speculate that the loss of PCGs expression in the sub-epidermal tissue could be due to the disorganization of the ECM. This was supported by the studies in vertebrate models, which showed that the ECM interaction with the tissue types was crucial for the regulation of gene expression (Boudreau et al., 1995; Spencer et al., 2007). However the crosstalk between ECM and sub-epidermis that is crucial for the regulation of PCG expression in planaria needs to be further investigated.

## DISCUSSION

Regeneration is a complex process mediated by stem cell function and tissue organization. Studies from hydra, amphibian and planaria have shown that non-stem cells express various tissue patterning genes essential for regeneration (Lengfeld et al., 2009; McCusker and Gardiner, 2014; Takahashi and Fujisawa, 2009; Witchley et al., 2013). However, the potential role of non-stem cells during regeneration remains largely unexplored. In this study, we show the pivotal role of epidermal integrity and the crucial role played by the RNA-binding protein PABPC2 in epithelialization and epidermal turnover during planarian regeneration and homeostasis.

*smed-pabpc2* is expressed in multiple cell types, except pharynx and terminally differentiated epidermal cells (*NB.22.1e^+^* and *laminB^+^*). Strikingly, *pabpc2* knockdown animals showed perturbation mainly in epidermal tissue despite its absence in the epidermal cells. This defect in the epidermal organization could be either due to the defect in the formation of the epidermal progenitors and/or maintenance of the epidermal integrity. Whole-mount *in situ* hybridization for epidermal progenitors on animals fed with a low dose of *pabpc2* dsRNA (1st feed) showed substantial reduction in the progenitor population. However, the other progenitors remained seemingly unaffected, even after 48 h post-amputation, suggesting that epidermal progenitors are exquisitely sensitive to *pabpc2* knockdown compared with the other progenitors. This result was also supported by the BrdU-labeling experiments, which showed failure in differentiation of neoblasts to epidermal progenitors. Furthermore, we also observed no detectable expression of *pabpc2* in epidermal cells, which suggests that PABPC2 might not be directly involved in the maintenance of epidermal tissue. Taken together, our results indicate that the defects observed in the epidermal tissue are most likely due to the defect in the epithelial lineage specification. However, it is difficult to rule out the direct role of PABPC2 in epidermal organization exclusively based on the lack of expression of *pabpc2 in NB.22.1e^+^ and laminB^+^* cells*.* Currently, the lack of markers to study various epidermal cell types limits our understanding regarding the expression of *pabpc2* in those cell types.

Epidermis is well characterized for its protective function. Upon amputation/injury, epidermal cells provide the first response by covering the wound within a few hours (Chandebois, 1980; Morita and Best, 1974). It has been shown that the epithelial cells near the site of injury come into direct contact to the underlying sub-epidermal muscle (Schurmann and Peter, 1998). In *pabpc2* knockdown animals, we observed the rounded appearance of the epithelial cells near the wound region, suggesting a defect in the attachment of the epithelial cells to the underlying sub-epidermal muscle. In addition, we also observed blistering of the epidermal tissue and the disorganization of the ECM in the regions away from the wound in the *pabpc2* knockdown animals. RNA-binding proteins such as PABPs have been implicated in formation of integrin-rich focal adhesion complexes that are essential for epidermis-ECM interactions (Babic et al., 2009; de Hoog et al., 2004; Lee et al., 2009). Depletion of these RNA-binding proteins alters cell morphology and the ability of the cell to spread post-adhesion (Chicurel et al., 1998; de Hoog et al., 2004; Katz et al., 2012). Thus, we speculate that the knockdown of *pabpc2* may result in the disruption of cell matrix adhesion, resulting in a defect in epithelialization. However, it is not clear whether the blistering defects observed are either a consequence of a defect in the epidermal turnover or a direct effect of *pabpc2* knockdown on the epidermal organization.

Furthermore, in *pabpc2* knockdown animals, we also observed prolonged expression of the early wound-response genes (*jun-1*, *fos-1*, *egrl1* and *notum*). In the regenerating animals, these genes are upregulated within 30 mpa to 3 hpa, and their expression is subsequently downregulated by 12 hpa (Wenemoser et al., 2012; Wurtzel et al., 2015). We believe the epidermal defect in the knockdown animals was perceived as a sustained injury leading to the upregulation and prolonged expression of the early wound-response genes. This was also supported by the enriched expression of wound-response genes *jun-1* and *fos-1* in *pabpc2* knockdown uncut animals. However, it is also possible that PABPC2 directly regulates expression of these wound-healing genes, which is currently difficult to address due to the lack of antibody.

Localized proliferation of neoblasts occurs only in the amputated animals near the site of injury (Wenemoser and Reddien, 2010). However, the factors essential for the switch from global to localized proliferation remains unknown. Lin and Pearson (2017) have shown that the prolonged expression of the early wound-response genes in *yorkie* knockdown animals affected the bimodal proliferation of neoblast. Yorkie is a transcriptional co-activator in the Hippo kinase cascade, the knockdown of which led to prolonged global proliferation of neoblasts and delayed second phase of proliferation, resulting in the defective blastema formation (Lin and Pearson, 2017). Similarly, knockdown of *follistatin*, an early wound-response gene, showed failure in second phase of neoblast proliferation and blastema formation (Gaviño et al., 2013; Roberts-Galbraith and Newmark, 2015). Together, these studies suggest a strong correlation between the regulation of wound response and proliferation of neoblasts crucial for blastema formation. In *pabpc2* knockdown animals, the sustained global neoblast proliferation and failure in localized proliferation could be an outcome of prolonged early wound response. The other possibility could be the downregulation of the intrinsic factors in the neoblasts upon *pabpc2* knockdown, resulting in the failure of localized proliferation.

Studies have shown that the localized proliferation is essential for blastema formation (Gaviño et al., 2013; Lin and Pearson, 2017; Roberts-Galbraith and Newmark, 2015). In *pabpc2* knockdown animals, failure in the neoblast proliferation near the wound region explains the defect in blastema formation, which was verified by the absence of progenitors in the blastema at 3 dpa. Our result suggests that the defect in the blastema could be a consequence of defect in epithelial-basement membrane attachments, which have been suggested to have an instructive role in planarian blastema formation (Reddien and Sanchez Alvarado, 2004).

The best characterized function of poly (A)-tail-bound PABPC is enhancing translation initiation by interacting with translation initiation factors bound at the 5′-end of the mRNA. Although considered as a ‘global’ effector of translation, the individual mRNAs that are translationally regulated vary among different PABPCs. For example, in *Xenopus*, the knockdown of the three different PABPCs showed distinct cellular and developmental phenotypes (Smith et al., 2014). Studies in *C. elegans* have also shown that out of the two PABP isoforms, PAB-1, but not PAB-2, is essential for fertility (Smith et al., 2014). This suggests that each PABPC has a specific set of targets that it regulates, although the factors that impart specificity are not well understood. RAR analysis in control and *pabpc2* knockdown animals revealed that PABPC2 regulates the translation of specific set of targets, such as *zfp-1*, *gata123* and *odc-1*, that are essential for epidermal lineage formation. Thus, these results highlight the pivotal role of PABPC2 in regulating the translation of the transcripts that are essential for the formation of epidermal progenitors in planarians, and this is crucial for the maintenance of epidermal organization.

**Fig. 6.**
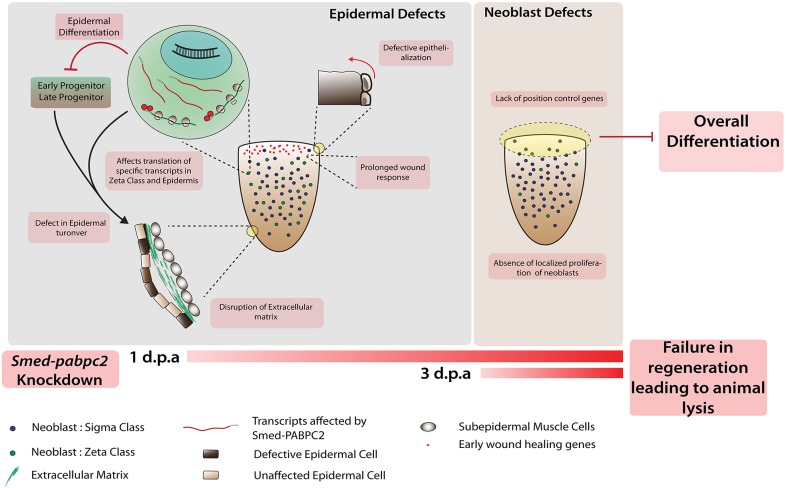
**Model showing the crucial role of *smed-pabpc2* in epidermal integrity and neoblast function.** *smed*-*pabpc2* knockdown animals show failure of epidermal organization due to failure of epidermal turnover. The epidermal defects leads to several other defects, such as loss of ECM integrity, defective wound closure and prolonged wound response. These potentially lead to a neoblast proliferation defect near the wound region and absence of PCGs, which subsequently affects overall differentiation and planarian regeneration.

The facts that the animals lyse within one week post-amputation and that PABPC is a multi-functional protein make it difficult to delineate the primary and secondary consequences. Given that epidermal defects manifest themselves much earlier than other tissues, it is most likely the primary consequence of *pabpc2* knockdown. Thus, we speculate that the epithelial phenotype might be the cause of all other defects, including the defect in localized proliferation of the neoblast (Fig. 6).

The cellular events that facilitate regeneration in planaria are comparable with the events crucial to tissue regeneration in higher metazoans. For example, the events essential for wound healing in planaria, such as epithelialization of the wound surface, are also observed during the cutaneous wound healing in vertebrates (Arwert et al., 2012; Jamora, 2014). Similarly, mobilization of the neoblast stem cells to the site of injury, which is crucial for planarian regeneration, has also been shown to be essential in metazoans (Arwert et al., 2012; Jamora, 2014). However, the possible influence of epidermal and ECM organization during regeneration has not been comprehensively investigated in higher metazoans. In the current study, we uncover a novel role for PABPC2 in epidermal turnover and organization, which is essential for planarian regeneration.

## MATERIALS AND METHODS

### Planarian culture

Animals used in this study belonged to the sexual strain of the species *Schmidtea mediterranea*. They were maintained at 20°C in planarian media (2 mM NaCl, 0.1 mM KCl, 0.1 mM MgSO_4_, 0.12 mM NaHCO_3_ in distilled water), and fed beef liver paste twice a week. Animals were starved one week prior to any experiments.

### RNAi experiments

RNAi was carried out using the feeding protocol described previously (Reddien et al., 2005). Post-feeding, animals were cut into three fragments: head, trunk and tail.

### Whole-mount *in situ* hybridization and double fluorescence *in situ* hybridization

Digoxigenin- or fluorescein-labeled RNA probes were synthesized using an *in vitro* transcription kit (Roche). Whole-mount in situ hybridization and double fluorescence in situ hybridization were carried out as described previously (Pearson et al., 2009; King and Newmark, 2013).

### Whole-mount immunostaining

Animals were treated in 2% HCl for 5 min on ice. They were then incubated in Carnoy's fixative for 2.5 to 3 h. One rinse in absolute methanol and overnight bleaching in 6% hydrogen peroxide followed fixation. The animals were then rehydrated in graded methanol PBS washes, and incubated in blocking solution (10% horse serum in PBSTx) for 6 h. Primary antibody (anti phospho-histone H3 ser10, Abcam 06-570; 6G10, DSHB; and rootletin antibody, a kind gift from Dr Jochen Rink, Max Planck Institute of Molecular Cell Biology and Genetics, Dresden, Germany) incubation was carried out overnight at room temperature at dilutions of 1:250 (rootletin antibody) or 1:100 (H3PS10 antibody). After four washes (2 h each) at room temperature with blocking solution, animals were incubated in secondary antibody solution (Alexa Fluor donkey anti mouse-488 and Alexa Fluor donkey anti rabbit-488, Molecular Probes) overnight at room temperature. For concavalin A-FITC staining, animals were incubated for 4 h at room temperature after blocking in Carbo-free blocking solution (Vector Labs) at a dilution of 1:2500. For 6G10 immunostaining, bleached animals were blocked in 10% horse serum in PBSTx blocking solution and were incubated with anti 6G10 antibody (diluted 1:5) overnight at 4°C.

### Image acquisition and quantitation

Images were acquired using the LSM 780 laser scanning confocal microscope. Quantitation of H3P-positive cells was carried out using the multiple pointer tool on ImageJ. RS/RAW quantitation was carried out by counting H3P-positive cells on both the regenerating as well as non-regenerating ends of the animal. The animals were divided in the ratio of 1:2 for RS and RAW, respectively, and the number of cells was counted in respective regions and was normalized to the body area. Colocalization of the different progenitor and tissue population *agat1*, *prog1*, *collagen*, *smedwi1*, *chat and porcupine* with *pabpc2* was quantified using MATLAB (Mathworks). (See supplementary Materials and Methods for details.)

### Embedding and cryosectioning

Regenerating fragments were treated with 2% HCl and fixed in Carnoy's fixative. The dehydrated animals were given two washes for 30 min in 100% xylene. Xylene was replaced by melted paraffin wax for 1 h at 70°C and the animals were embedded in paraffin wax in commercially available molds (Tissue-Tek, 4566). Animals were subsequently sectioned sagittally at 16 µm using a Cryostat Leica 1850.

### Immunostaining on sections

Slides containing planarian sections were fixed with 4% formaldehyde for 20 min. They were washed thrice in PBS and subsequently blocked for 1 h in 10% horse serum in PBSTx (PBS+0.3% Triton-X). Overnight incubation with primary antibody was performed at 4°C (rabbit anti-mouse collagen IV, Abcam, ab-756p; 1:200). Secondary antibody was incubated for 45 min (Alexa-Fluor anti-rabbit 488, Molecular Probes). Sections were then washed and stained with DAPI.

### Transmission electron microscopy

Samples were prepared as described previously (Brubacher et al., 2014). N-block staining using 2% urnayl acetate was carried out for 1 h prior to dehydration with graded ethanol series. Ultrathin sections of 60 nm were cut using an RMC cryo ultra-microtome equipped with a diamond knife; sections were then placed on 200 mesh copper grids. Stained sections were observed with a TEM (Tecnai G^2^ Spirit Bio-TWIN) operating at 100 kV. The electron micrographs were digitized using Adobe Photoshop by adjusting the contrast and the brightness balance.

### Polysome profiling

Animals were soaked in cycloheximide (CHX) for 2 h at 10 µg/ml concentration in planaria media. They were than macerated in cold CMF (Ca^2+^/Mg^2+^-free media) with CHX followed by collagenase treatment for 30 min to dissociate them into cells. The cells were than pelleted and washed three times with CMF+CHX and resuspended in 500 µl of lysis buffer (6 M urea, 2 M thiourea, 50 mM DTT, 5% glycerol, 1× PIC, RNAse inhibitor and CHX). Cells were incubated in lysis buffer for 30 min at 4°C and then vigorously pipetted up and down for complete lysis of cells. Planarian lysate was loaded onto a sucrose gradient (15%-45%) and centrifuged at 276,960 ***g*** for 2 h. The sucrose gradient was then loaded on to an ISCO Teledyne Gradient fractionator. Sixty percent sucrose was pumped from below to push the sucrose gradient to the UV chamber. Constant absorbance was measured and the fractions were collected.

### qRT-PCR analysis

Total RNA from samples of five or six animals was extracted with Trizol reagent (Invitrogen) and cDNAs were synthesized with SuperScriptII Reverse Transcriptase (Invitrogen). qRT-PCR experiments were then performed using the Absolute qRT-PCR SYBR Green Master Mix (Thermo Scientific). Experiments were performed on three biological replicates per time point. Each biological replicate was technically replicated three times in each reaction. *smed-actin* was used for normalization. Students *t*-test was used to test for statistical significance.

Following primers were used for qRT-PCR: *smed-jun1*, 5′-TCCAGTAACCAGCCACAACT-3′ and 5′-AAAGCGCGTTGTTTTCTTGT-3′; *smed-fos1*, 5′-CCGGTAACTGCAACTAAGCC-3′ and 5′-ACTGAAATTGGCGTCGTTCA-3′; *smed-traf1*, 5′-AATAGTGTGGCCGTTTCGAC-3′ and 5′-GGCTGACCTGCTCCTACATT-3′; *smed-egr3*, 5′-TCGTCGGGATGAATTGAAAAGA-3′ and 5′-ATGTCGCAACCTTTCGTCTG-3′; *smed-notum*, 5′-AAACCGGCAAGTCTCCATGT-3′ and 5′-TGGGAAAGCGGTGAACATGT-3′; *smed-zfp-1*, 5′-AGCCAAAATAGTCCAGTACCCA-3′ and 5′-TGGTGTTGATTTTCGCTTCTGT-3′.

### mRNA purification and transcriptome sequencing

Total RNA was purified using TRIzol reagent from the desired polysome fraction and also from the whole animal, and sequenced using Illumina TrueSeq RNA Samole preparation kit v2 on HiSeq. Details of RNA-seq analysis can be found in the supplementary Materials and Methods.

### BrdU labeling and immunofluorescence

10 mg/ml BrdU solution was prepared in 1× planaria media containing 10% DMSO, which was injected in planarians. BrdU staining was carried out as described previously (Vasquez-Doorman and Petersen, 2014) with few modifications. After fluorescence *in situ* hybridization development as described previously (King and Newmark, 2013), POD was inactivated with 2N HCl for 45 min, followed by antibody labeling of BrdU. Rat anti-BrdU (Abcam) was used at 1:1000 dilution.

## References

[DEV152942C1] Aboobaker A. A. (2011). Planarian stem cells: a simple paradigm for regeneration. *Trends Cell Biol.* 21, 304-311. 10.1016/j.tcb.2011.01.005 21353778

[DEV152942C2] Arwert E. N., Hoste E. and Watt F. M. (2012). Epithelial stem cells, wound healing and cancer. Nature reviews. *Cancer* 12, 170-180. 10.1038/nrc3217 22362215

[DEV152942C3] Babic I., Sharma S. and Black D. L. (2009). A role for polypyrimidine tract binding protein in the establishment of focal adhesions. *Mol. Cell. Biol.* 29, 5564-5577. 10.1128/MCB.00590-09 19667078 PMC2756873

[DEV152942C4] Baguñà J. (2012). The planarian neoblast: the rambling history of its origin and some current black boxes. *Int. J. Dev. Biol.* 56, 19-37. 10.1387/ijdb.113463jb 22252540

[DEV152942C5] Boudreau N., Myers C. and Bissell M. J. (1995). From laminin to lamin: regulation of tissue-specific gene expression by the ECM. *Trends Cell Biol.* 5, 1-4. 10.1016/S0962-8924(00)88924-2 14731421

[DEV152942C6] Brubacher J. L., Vieira A. P. and Newmark P. A. (2014). Preparation of the planarian Schmidtea mediterranea for high-resolution histology and transmission electron microscopy. *Nat. Protoc.* 9, 661-673. 10.1038/nprot.2014.041 24556788 PMC4097191

[DEV152942C7] Cebrià F., Vispo M., Newmark P., Bueno D. and Romero R. (1997). Myocyte differentiation and body wall muscle regeneration in the planarian Girardia tigrina. *Dev. Genes Evol.* 207, 306-316. 10.1007/s004270050118 27747428

[DEV152942C8] Chandebois R. (1980). The dynamics of wound closure and its role in programming of planarian regeneration II- Distalization. *Dev. Growth Differ.* 22, 693-704. 10.1111/j.1440-169X.1980.00693.x 37281333

[DEV152942C9] Chicurel M. E., Singer R. H., Meyer C. J. and Ingber D. E. (1998). Integrin binding and mechanical tension induce movement of mRNA and ribosomes to focal adhesions. *Nature* 392, 730-733. 10.1038/33719 9565036

[DEV152942C10] de Hoog C. L., Foster L. J. and Mann M. (2004). RNA and RNA binding proteins participate in early stages of cell spreading through spreading initiation centers. *Cell* 117, 649-662. 10.1016/S0092-8674(04)00456-8 15163412

[DEV152942C11] Forsthoefel D. J., James N. P., Escobar D. J., Stary J. M., Vieira A. P., Waters F. A. and Newmark P. A. (2012). An RNAi screen reveals intestinal regulators of branching morphogenesis, differentiation, and stem cell proliferation in planarians. *Dev. Cell* 23, 691-704. 10.1016/j.devcel.2012.09.008 23079596 PMC3521571

[DEV152942C12] Gaviño M. A., Wenemoser D., Wang I. E. and Reddien P. W. (2013). Tissue absence initiates regeneration through follistatin-mediated inhibition of activin signaling. *Elife* 2, e00247 10.7554/eLife.00247 24040508 PMC3771573

[DEV152942C13] Gorgoni B., Richardson W. A., Burgess H. M., Anderson R. C., Wilkie G. S., Gautier P., Martins J. P. S., Brook M., Sheets M. D. and Gray N. K. (2011). Poly(A)-binding proteins are functionally distinct and have essential roles during vertebrate development. *Proc. Natl. Acad. Sci. USA* 108, 7844-7849. 10.1073/pnas.1017664108 21518916 PMC3093506

[DEV152942C14] Goss D. J. and Kleiman F. E. (2013). Poly(A) binding proteins: are they all created equal? Wiley interdisciplinary reviews. *RNA* 4, 167-179. 10.1002/wrna.1151 23424172 PMC3580857

[DEV152942C15] Guo T., Peters A. H. and Newmark P. A. (2006). A Bruno-like gene is required for stem cell maintenance in planarians. *Dev. Cell* 11, 159-169. 10.1016/j.devcel.2006.06.004 16890156

[DEV152942C16] Gurley K. A., Elliott S. A., Simakov O., Schmidt H. A., Holstein T. W. and Sanchez Alvarado A. (2010). Expression of secreted Wnt pathway components reveals unexpected complexity of the planarian amputation response. *Dev. Biol.* 347, 24-39. 10.1016/j.ydbio.2010.08.007 20707997 PMC2966944

[DEV152942C17] Hori I. (1979). Regeneration of the epidermis and basement membrane of the planarian Dugesia japonica after total-body X irradiation. *Radiat. Res.* 77, 521-533. 10.2307/3575163 441256

[DEV152942C18] Hori I. (1980). Localization of newly synthesized precursors of basal lamina in the regenerating planarian as revealed by autoradiography. *Tissue Cell* 12, 513-521. 10.1016/0040-8166(80)90040-3 7434335

[DEV152942C19] Hori I. (1991). Differentiation of epidermal cells in the regenerating planarian Dugesia japonica. *Hydrobiologia* 227, 19-24. 10.1007/BF00027576

[DEV152942C20] Jamora C. (2014). Mechanisms of wound repair. In *Stem Cells: From Basic Research to Therapy. Tissue Homeostasis and Regeneration During Adulthood, Applications Legislation and Ethics* (ed. Calegari, F. and Waskow, C.) Vol. 2, pp. 67-103.

[DEV152942C21] Katz Z. B., Wells A. L., Park H. Y., Wu B., Shenoy S. M. and Singer R. H. (2012). beta-Actin mRNA compartmentalization enhances focal adhesion stability and directs cell migration. *Genes Dev.* 26, 1885-1890. 10.1101/gad.190413.112 22948660 PMC3435492

[DEV152942C22] King R. S. and Newmark P. A. (2013). In situ hybridization protocol for enhanced detection of gene expression in the planarian Schmidtea mediterranea. *BMC Dev. Biol.* 13, 8 10.1186/1471-213X-13-8 23497040 PMC3610298

[DEV152942C23] Lakshmanan V., Bansal D., Kulkarni J., Poduval D., Krishna S., Sasidharan V., Anand P., Seshasayee A. and Palakodeti D. (2016). Genome-wide analysis of polyadenylation events in schmidtea mediterranea. *G3 (Bethesda)* 6, 3035-3048. 10.1534/g3.116.031120 27489207 PMC5068929

[DEV152942C24] Lee J. H., Rangarajan E. S., Yogesha S. D. and Izard T. (2009). Raver1 interactions with vinculin and RNA suggest a feed-forward pathway in directing mRNA to focal adhesions. *Structure* 17, 833-842. 10.1016/j.str.2009.04.010 19523901 PMC2811071

[DEV152942C25] Lengfeld T., Watanabe H., Simakov O., Lindgens D., Gee L., Law L., Schmidt H. A., Ozbek S., Bode H. and Holstein T. W. (2009). Multiple Wnts are involved in Hydra organizer formation and regeneration. *Dev. Biol.* 330, 186-199. 10.1016/j.ydbio.2009.02.004 19217898

[DEV152942C26] Lin A. Y. T. and Pearson B. J. (2017). Yorkie is required to restrict the injury responses in planarians. *PLoS Genet.* 13, e1006874 10.1371/journal.pgen.1006874 28686611 PMC5515462

[DEV152942C27] Mangus D. A., Evans M. C. and Jacobson A. (2003). Poly(A)-binding proteins: multifunctional scaffolds for the post-transcriptional control of gene expression. *Genome Biol.* 4, 223 10.1186/gb-2003-4-7-223 12844354 PMC193625

[DEV152942C28] Martin G. G. (1978). A new function of rhabdites: mucus production for ciliary gliding. *Zoomorphologie* 91, 235-248. 10.1007/BF00999813

[DEV152942C29] McCusker C. D. and Gardiner D. M. (2014). Understanding positional cues in salamander limb regeneration: implications for optimizing cell-based regenerative therapies. *Dis. Models Mech.* 7, 593-599. 10.1242/dmm.013359 PMC4036467 24872456

[DEV152942C30] Mii Y. and Taira M. (2009). Secreted Frizzled-related proteins enhance the diffusion of Wnt ligands and expand their signalling range. *Development* 136, 4083-4088. 10.1242/dev.032524 19906850

[DEV152942C31] Morita M. and Best J. B. (1974). Electron microscopic studies of planarian regeneration. II. Changes in epidermis during regeneration. *J. Exp. Zoolog.* 187, 345-373. 10.1002/jez.1401870305 4820343

[DEV152942C32] Morita M., Best J. B. and Noel J. (1969). Electron microscopic studies of planarian regeneration. I. Fine structure of neoblasts in Dugesia dorotocephala. *J. Ultrastruct. Res.* 27, 7-23. 10.1016/S0022-5320(69)90017-3 5769728

[DEV152942C33] Palakodeti D., Smielewska M., Lu Y.-C., Yeo G. W. and Graveley B. R. (2008). The PIWI proteins SMEDWI-2 and SMEDWI-3 are required for stem cell function and piRNA expression in planarians. *RNA* 14, 1174-1186. 10.1261/rna.1085008 18456843 PMC2390803

[DEV152942C34] Pearson B. J., Eisenhoffer G. T., Gurley K. A., Rink J. C., Miller D. E., Sanchez, and Alvarado A. (2009). Formaldehyde-based whole-mount in situ hybridization method for planarians. *Dev. Dyn.* 238, 443-450. 10.1002/dvdy.21849 19161223 PMC2640425

[DEV152942C35] Petersen C. P. and Reddien P. W. (2011). Polarized notum activation at wounds inhibits Wnt function to promote planarian head regeneration. *Science* 332, 852-855. 10.1126/science.1202143 21566195 PMC3320723

[DEV152942C36] Reddien P. W. and Sanchez Alvarado A. (2004). Fundamentals of planarian regeneration. *Annu. Rev. Cell Dev. Biol.* 20, 725-757. 10.1146/annurev.cellbio.20.010403.095114 15473858

[DEV152942C37] Reddien P. W., Bermange A. L., Murfitt K. J., Jennings J. R. and Sánchez Alvarado A. (2005). Identification of genes needed for regeneration, stem cell function, and tissue homeostasis by systematic gene perturbation in planaria. *Dev. Cell* 8, 635-649. 10.1016/j.devcel.2005.02.014 15866156 PMC2267917

[DEV152942C38] Reddien P. W., Bermange A. L., Kicza A. M. and Sanchez Alvarado A. (2007). BMP signaling regulates the dorsal planarian midline and is needed for asymmetric regeneration. *Development* 134, 4043-4051. 10.1242/dev.007138 17942485

[DEV152942C39] Resch A. M., Palakodeti D., Lu Y.-C., Horowitz M. and Graveley B. R. (2012). Transcriptome analysis reveals strain-specific and conserved stemness genes in Schmidtea mediterranea. *PloS ONE* 7, e34447 10.1371/journal.pone.0034447 22496805 PMC3319590

[DEV152942C40] Rink J. C. (2013). Stem cell systems and regeneration in planaria. *Dev. Genes Evol.* 223, 67-84. 10.1007/s00427-012-0426-4 23138344 PMC3552358

[DEV152942C41] Rink J. C., Vu H. T.-K. and Sanchez Alvarado A. (2011). The maintenance and regeneration of the planarian excretory system are regulated by EGFR signaling. *Development* 138, 3769-3780. 10.1242/dev.066852 21828097 PMC3152929

[DEV152942C42] Roberts-Galbraith R. H. and Newmark P. A. (2015). On the organ trail: insights into organ regeneration in the planarian. *Curr. Opin. Genet. Dev.* 32, 37-46. 10.1016/j.gde.2015.01.009 25703843

[DEV152942C43] Ross K. G., Omuro K. C., Taylor M. R., Munday R. K., Hubert A., King R. S. and Zayas R. M. (2015). Novel monoclonal antibodies to study tissue regeneration in planarians. *BMC Dev. Biol.* 15, 2 10.1186/s12861-014-0050-9 25604901 PMC4307677

[DEV152942C44] Rouhana L., Shibata N., Nishimura O. and Agata K. (2010). Different requirements for conserved post-transcriptional regulators in planarian regeneration and stem cell maintenance. *Dev. Biol.* 341, 429-443. 10.1016/j.ydbio.2010.02.037 20230812

[DEV152942C45] Sasidharan V., Lu Y. C., Bansal D., Dasari P., Poduval D., Seshasayee A., Resch A. M., Graveley B. R. and Palakodeti D. (2013). Identification of neoblast- and regeneration-specific miRNAs in the planarian Schmidtea mediterranea. *RNA* 19, 1394-1404. 10.1261/rna.038653.113 23974438 PMC3854530

[DEV152942C46] Schurmann W. and Peter R. (1998). Inhibition of regeneration in the planarian Dugesia polychroa (Schmidt) by treatment with magnesium chloride: a morphological study of wound closure. *Hydrobiologia* 383, 111-116.

[DEV152942C47] Scimone M. L., Srivastava M., Bell G. W. and Reddien P. W. (2011). A regulatory program for excretory system regeneration in planarians. *Development* 138, 4387-4398. 10.1242/dev.068098 21937596 PMC3177309

[DEV152942C48] Scimone M. L., Kravarik K. M., Lapan S. W. and Reddien P. W. (2014). Neoblast specialization in regeneration of the planarian Schmidtea mediterranea. *Stem Cell Rep.* 3, 339-352. 10.1016/j.stemcr.2014.06.001 PMC4176530 25254346

[DEV152942C49] Skaer R. J. (1965). The origin and continuous replacement of epidermal cells in the planarian Polycelis tenuis (Iijima). *J. Embryol exp Morph* 13, 129-139. 14296272

[DEV152942C50] Smith R. W. P., Blee T. K. P. and Gray N. K. (2014). Poly(A)-binding proteins are required for diverse biological processes in metazoans. *Biochem. Soc. Trans.* 42, 1229-1237. 10.1042/BST20140111 25110030 PMC4128646

[DEV152942C51] Solana J., Gamberi C., Mihaylova Y., Grosswendt S., Chen C., Lasko P., Rajewsky N. and Aboobaker A. A. (2013). The CCR4-NOT complex mediates deadenylation and degradation of stem cell mRNAs and promotes planarian stem cell differentiation. *PLoS Genet.* 9, e1004003 10.1371/journal.pgen.1004003 24367277 PMC3868585

[DEV152942C52] Spencer V. A., Xu R. and Bissell M. J. (2007). Extracellular matrix, nuclear and chromatin structure, and gene expression in normal tissues and malignant tumors: a work in progress. *Adv. Cancer Res.* 97, 275-294. 10.1016/S0065-230X(06)97012-2 17419950 PMC2912285

[DEV152942C53] Takahashi T. and Fujisawa T. (2009). Important roles for epithelial cell peptides in hydra development. *BioEssays* 31, 610-619. 10.1002/bies.200800163 19382229

[DEV152942C54] Tarun S. Z. Jr. and Sachs A. B. (1996). Association of the yeast poly(A) tail binding protein with translation initiation factor eIF-4G. *EMBO J.* 15, 7168-7177. 9003792 PMC452544

[DEV152942C55] Tazaki A., Kato K., Orii H., Agata K. and Watanabe K. (2002). The body margin of the planarian Dugesia japonica: characterization by the expression of an intermediate filament gene. *Dev. Genes Evol.* 212, 365-373. 10.1007/s00427-002-0253-0 12203092

[DEV152942C56] Tu K. C., Cheng L. C., T K Vu H, Lange J. J., McKinney S. A., Seidel C. W. and Sanchez Alvarado A. (2015). Egr-5 is a post-mitotic regulator of planarian epidermal differentiation. *Elife* 4, e10501 10.7554/eLife.10501 26457503 PMC4716842

[DEV152942C57] van Wolfswinkel J. C., Wagner D. E. and Reddien P. W. (2014). Single-cell analysis reveals functionally distinct classes within the planarian stem cell compartment. *Cell Stem Cell* 15, 326-339. 10.1016/j.stem.2014.06.007 25017721 PMC4171737

[DEV152942C58] Vasquez-Doorman C. and Petersen C. P. (2014). zic-1 Expression in Planarian neoblasts after injury controls anterior pole regeneration. *PLoS Genet.* 10, e1004452 10.1371/journal.pgen.1004452 24992682 PMC4081000

[DEV152942C59] Wagner D. E., Wang I. E. and Reddien P. W. (2011). Clonogenic neoblasts are pluripotent adult stem cells that underlie planarian regeneration. *Science* 332, 811-816. 10.1126/science.1203983 21566185 PMC3338249

[DEV152942C60] Wang Y., Stary J. M., Wilhelm J. E. and Newmark P. A. (2010). A functional genomic screen in planarians identifies novel regulators of germ cell development. *Genes Dev.* 24, 2081-2092. 10.1101/gad.1951010 20844018 PMC2939369

[DEV152942C61] Wenemoser D. and Reddien P. W. (2010). Planarian regeneration involves distinct stem cell responses to wounds and tissue absence. *Dev. Biol.* 344, 979-991. 10.1016/j.ydbio.2010.06.017 20599901 PMC2950745

[DEV152942C62] Wenemoser D., Lapan S. W., Wilkinson A. W., Bell G. W. and Reddien P. W. (2012). A molecular wound response program associated with regeneration initiation in planarians. *Genes Dev.* 26, 988-1002. 10.1101/gad.187377.112 22549959 PMC3347795

[DEV152942C63] Witchley J. N., Mayer M., Wagner D. E., Owen J. H. and Reddien P. W. (2013). Muscle cells provide instructions for planarian regeneration. *Cell Rep.* 4, 633-641. 10.1016/j.celrep.2013.07.022 23954785 PMC4101538

[DEV152942C64] Wolff E. (1962). *Recent Researches on the Regeneration of Planaria*. New York: Ronald Press.

[DEV152942C65] Wurtzel O., Cote L. E., Poirier A., Satija R., Regev A. and Reddien P. W. (2015). A Generic and Cell-Type-Specific Wound Response Precedes Regeneration in Planarians. *Dev. Cell* 35, 632-645. 10.1016/j.devcel.2015.11.004 26651295 PMC4817857

[DEV152942C66] Zayas R. M., Cebrià F., Guo T., Feng J. and Newmark P. A. (2010). The use of lectins as markers for differentiated secretory cells in planarians. *Dev. Dyn.* 239, 2888-2897. 10.1002/dvdy.22427 20865784 PMC3004010

